# Gynæcological Medicine

**Published:** 1871-05

**Authors:** T. S. Powell, W. T. Goldsmith


THE GEORGIA

### MEDICAL COMPANION:

A MONTHLY ADVISER.

VOL. I. ATLANTA, GA., MAY, 1871. No. 5.

### PART I.

### Original Communications and Special Selections.

### GYNÆCOLOGICAL MEDICINE.

*Report of the Committee of the Seventh Congressional District of Georgia, upon "Gynæcological Medicine," prepared to be read before the Georgia Medical Association. By T. S. Powell, M. D., and W. T. Goldsmith, M. D.*

In the broadest sense of the term—"Gynæcological Medicine"—upon which it has been made, by resolution of this Association, our duty to report, would require for its elaboration more time and space than we could possibly devote to it, or this body give for its consideration.

The object of the resolution, appointing a Committee from each Congressional District of the State, to report upon "Gynæcological Medicine," was intended, doubtless, to have the different Committees report upon those diseases of females, and the treatment best adopted for their alleviation and cure, of most frequent occurrence in their various sections, and not to encroach upon the field of speculative inquiry in regard to Gynæcological discussion. Believing this to be the object, we shall at once, address ourselves to the consideration of those diseases coming most commonly under our observation. We will, therefore, confine our remarks to the diseases of the uterus proper—under the anatomical divisions of cervix and body—cervical canal, and uterine cavity, and its os externum and os internum.

In entering upon the study and treatment of uterine diseases, it is important to keep in mind a few facts, upon which must rest all prospect for their successful management:

First—The anatomical structures forming the uterus, its appendages and attachments; Secondly—An accurate conception, as far as possible, of their relations to other and distant organs, through the great sympathetic, and other nerves; the normal physiological functions of the uterine organs; and lastly, the true position of the uterus in its pregnated and unimpregnated condition, and the respective relations of the organ to the different structures and parts within the pelvis, by which it is surrounded. The great practical importance and value of the advice of the late Prof. Meiggs, should be ever present in the minds of all, to wit: To have constantly in view a true ideal—a fixed standard—of the healthy uterus—its form, size, color and position in relation to the vagina and other parts, and their true place in the pelvic cavity. Without an acurate knowledge of the anatomical structures composing these organs and parts, as well as the physiological functions which they perform in health, no substantial uterine pathology can be established, and no scientific treatment determined for the successful treatment of their diseases. No less acurate must be the knowledge of the physiological changes to which they are constantly subjected—changes frequently so different in different persons, and often, at times, in the same individual—in order to determine the fact of even the existence of disease. What in some individuals would be properly considered a healthy and normal condition, would amount to diseased action, requiring remedial measures for restoration to a normal condition in others; and a want of information as to the existence of these differences among females, and a practical knowedge of their diseases have, doubtless, been the true source of the many and various conflicting systems of treatment and misapplication of remedial agents. The one to whom success is most frequently awarded is, no doubt, the pains-taking, acurate and practical gynæcologist—one whose opinions are not bound to any exclusive system, but who draws upon all for the treatment best adapted to each particular case.

The first pathological change resulting in disease which we shall notice, is *congestion of the cervix uteri.* At every menstrual period there is, in woman, a physiological congestion of the uterus. This is a normal, healthy congestion, when healthfully performed, and is the type of the diseased condition under consideration, being a pathological change from a true, physiological function, not intermittent, but continuous. In order to determine this pathological change we appeal to symptoms as evidence, and to physical examination as proof. The latter we shall notice first. The finger being introduced, per varginam, properly eductated, as to touch, we bear in mind the normal, healthy standard—the "ideal." The cervix in this condition, is found soft, smooth and of proper size with its os normal; upon being pressed by the point of the finger, it transmits no sensation of hardness or resistence; is elastic and unctious, giving no pain when suddenly touched, and if unimpregnated, moves before the pressure applied. The vagina is then carefully explored, in order to determine the existence or non-existence of induration in, or around, the lips of the os; or of adhesions, as well as the true position of the uterus to the vagina and the adjacent organs and parts. If there be mal-position, in order to arrive at its nature with accuracy, the rectum is explored by the finger, the bladder by the catheter, and the sound or uterine probe passed into the uterus.

When *congestion of the mucous membrane* occurs, the finger impinges upon an enlarged and tumified cervix, slightly depressed in the cavity of the pelvis, spungy, softer and more unctious than natural, and transmitting pain upon pressure. The speculum being introduced exhibits the cervix of deeper color than that of health, verging from a bright arterial to a darker venous tinge, as the congestion is more or less intense. The muciperous glands are enlarged and swollen, the os bathed with a more tenaceous mucous than that of health, and dotted with the red and white congested points of the follicles and their mucous secretions.

This is the most common and the mildest disease of the cervix to which females are subject. Like the higher grades of other pathological conditions of the cervix, it induces a leucorrhœal discharge, perhaps oftener than uterine catarrh; while displacements with dysmenorrhœa are the frequent concomitants arising from this cause. Married woman are most liable to its invasion, but virgins and unmarried females are not, by any means, exempt, and it is of occasional occurrence in those who are pregnant.

The causes of cervical congestions are various. It not unfrequently arrises from exposure to cold shortly before or immediately after a menstrual period, in which condition the uterus, as before stated, is more or less congested, and extremely liable of being rendered permanently so by exposure to atmospheric changes. It is caused frequently by pregnancy, child-bearing and excessive coition; by the presence of polypi, foreign bodies, and irritating injections. It is caused too, according to our experience, by frequent cold-water injections used immediately subsequent to the excitement and partial congestions of the parts attending sexual intercourse, with the view of preventing procreation. We are persuaded this cause is, perhaps, much more common than supposed.

*Inflammation of the cervix* presents all the characteristics of the milder condition of congestion in a more intense and aggravated form. The cervix in this condition, is swollen and red; the os uteri distended with muco-pus; the muciperous glands hypertrophied; the os wide and open or patulous; the lining membrane of the cervix of a dark and livid hue, bleeding readily when touched, especially if excoriations and ulcerations have occurred. The canal is more or less filled with muco-pus, difficult of removal, adhering closely to the membrane, which is not produced by simple congestion—which, according to Dr. Bennett, is proof of inflammatory action—or if no pus is present, a glairly, albuminous mucous obstructs the canal, protrudes from the os, being always the concomitant of inflammatory action of the cervix, and pathognominic of that condition.

The general symptoms of congestion and inflamation of the cervix are similar—differing in degree only. In congestion they are often, at first, slight. The patient discovers an increased mucous discharge issuring from the parts, and is troubled, occasionally with backache. As it advances to the inflammatory stage the flow of mucous increases and the pain becomes severer, both in the back and ovaries; there is a feeling of pressure, weight and dragging in the pelvis, aggravated by standing or walking; there is weakness, lassitude, with nervousness, assuming, occasionally, various mild forms of hysteria. The appetite declines, and the bowels become constipated. The menstrual function is more or less impaired, becoming less in quantity and frequently supplanted by the characteristic leucorrhœal discharge. At every menstrual period the pain increases, and when it continues throughout the entire period, is severe. There is often sickness and nausea, and the abdomen tender—especially over the ovaries.

We will next notice *granular ulceration of the os uteri.* This pathological condition which was known and described by the old authors, under the names of erosions, epithelial abrasions, etc., of the cervix, we find occurring in practice, as its modern name implies in the form of abrasions, with granulations on the labia and internal surface of the mucus membrane of the cervix uteri. The posterior lip of the os uteri is more frequently affected by this granular condition, than the anterior. It should be borne in mind, however, that all ulcerations of the os uteri at one stage are granular, being one of the processes of inflammatory action towards resolution. That the posterior lip should so often be diseased ; while the anterior escapes, is, no doubt, attributable, in part, to the constant flow of the acrid secretions of the canal passing over it, which presupposes a diseased condition of this part, occurring in connection with the primary affection. When both lips are equally affected, it is very frequently due, no doubt, to the concurrent results of acute or chronic inflammation, which, at its inception, presents the appearance of a simple, abraded surface, free from discharge—which is the first stage of that pathological condition, merging upon ulceration. A granular condition of the cervix is often discovered, with or without erosions, and is usually accompanied with vaginitis—general or localized in distinct patches. We find the vagina seldom exempt from the evidences of inflammation, when granular ulceration of the os exists, which is probably due to the acrid secretions issuing from the os uteri, proceeding from the cervical canal; and this affection is no doubt frequently the effects of a diseased action of the lining mucus membrane of the cervical canal and cavity of the uterus. Whether this be true or not, we still have reason to suspect that in many instances our success in the treatment of granular ulceration has been largely due to this suspicion of its origin. This suspicion as to its origin need not interfere with the employment of suitable remedies for the relief of what might be considered the result of diseased action elsewhere, and as soon as possible, giving attention to the parts diseased. When ulceration of the os uteri is the result of inflammation of the cervical tissue composing it, and produced from whatever cause, then the means employed to cure, may remove the cause inducing it, and may never be known or recognized; but if ulceration of the os uteri, and inflammation of the vagina be caused by discharges from the cervical canal or cavity of the uterus, it may be palliated, but not cured, until these diseased conditions are removed.

In *granular ulceration*, the os uteri is generally patulous. The finger may often be passed completely within it, sometimes only its point, while in all cases the os is usually more patulous than natural. It is important always to examine the inner surface of the cervical canal; and before passing on, it may be proper to briefly give the methods by which this may be accomplished. This may be satisfactorily done in almost every instance by using bright sunlight—or, if circumstances will not permit, bright artificial light—and throwing it through the speculum. The cervix, in these cases, is unusually large—too large, frequently to be embraced within the end of the cylindrical speculum. The os uteri, by the instrument, is converted by its pressure, into a prolonged slit. If the labia of the os be gently drawn asunder, and the anterior lip hooked up and elevated a sufficient extent of the cervical canal will be frequently revealed to enable us to determine whether or not the part is diseased. Another method for determining its existence, is by the endoscope, an instrument which we regard of no very great value in solving the various problems of uterine pathology, but by which it is claimed every portion of the cavity of the uterus may be explored, and its condition accurately ascertained. In unskillful and inexperienced hands, it is not free from serious objections.

We meet with this form of ulceration most commonly in females from 22 to 35 years of age, and not at the so-called critical time of life, as some have supposed. At this age, the exciting causes largely predominate; there is more activity and vitality of the parts; greater excesses in sexual indulgence, and abortion and child-bearing more common.

While it is unpleasant, were it the habit of physicians to examine all cases with the speculum, as soon as their female patients observe or complain of the appearance of discharges from the vagina, we doubt not but that strict attention to regimen and rest, proper ablution and vaginal injections, (as chlorate of potash,) would, in many instances, be cured without resorting to topical applications, and cauterizations. While applications of caustics, in a few cases, may sometimes have proven dangerous, as has been asserted by some authors, or even attended by fatal results when carelessly used, either in the application or under circumstances where they were counter-indicated, yet according to our experience the majority of them may be employed with great benefit and perfect safety. Although cauterization is not always necessary, where simple redness of the os uteri exists, still we have found it when properly applied, of great value. One reason, perhaps, why we have not seen any bad effects from their application, may be due to the fact that we never employ them where much pain of the parts exists; and very cautiously in cases of positive ulcerations, accompanied with great tumefaction, engorgement or hypertrophy. In these latter conditions caustics do harm, according to our experience, when used with a view of destroying the structures; and, although in such cases we almost invariably employ them, it is not for the purpose of cauterization, but that by gentle touches, their stimulating and alterative effects may change the character of the diseased parts, and arouse them to healthy action. We have found it important, in many cases, to accomplish this purpose by applying these remedial agents not only lightly, but to very small surfaces. In order to do this effectually, it is necessary to introduce a speculum of proper size, so as to receive the cervix alone within its open extremity; and should the upper portions of the vagina fall within the presenting part of the instrument, unless we wish these parts to receive the alterative effects of the caustic, to cover all that portion of the vagina—so exposed with pledgets of lint, that the applications may be carried alone to the diseased os and canal of the cervix; and we also place a pellet of cotton saturated with glycerine to the os—first attaching a string to the cotton for its removal. In all cases before using applications, the cervix should be gently freed of its mucus, which is frequently difficult to accomplish, from the tenacity of its adhesion to the mouths of the glands from which it is secreted. Small bits of soft sponge, or pellets of cotton, after being dipped in water, may be held in the speculum forceps, and gently used in wiping it away, and a stream of water afterwards thrown by a syringe upon the os. It should be borne in mind that these granular ulcerations sometimes bleed freely, unless the gentlest handling is employed, and always with deleterious results if the parts be seriously injured.

Sometimes, in women who have borne children, it is difficult to engage the os in the speculum, from the fact that the uterus is frequently lower than normal in the pelvis. The finger should be introduced first in all cases, to ascertain the size and position of the uterus in the pelvic cavity. When the os is much diseased, even the finger may, by being rudely introduced, cause bleeding from the parts, and obscure the os from view, when the speculum follows such examination. Even with the greatest care, the speculum will often wound the tender granulations and cause hemorrhage. Not unfrequently, if proper caution is not exercised, the folds of the vagina, which often simulate with fallacious exactnes, the cervix and its os, will receive the applications, instead of the parts supposed or intended, doing no good and sometimes manifest injury.

Belonging to this genus of ulcer, we frequently find a varicose condition of the os and cervix uteri—known as the *varicose, fungus or bleeding ulcer*, and so described by various authors. It is simply an elongation, and properly speaking, a hypertrophied condition of the papillæ, unprotected by epithelium. The cervix is, in this form of ulceration, enlarged and indurated, and when observed, found to be traversed in many directions, by dark-colored trunks, elevated above the surrounding surface, and inosculating one with another. At one of these points ulceration begins, extending through the coats of the vessels, forming an outlet for the escape of blood which at once ensues. It is livid, of irregular margins, and secretes pus freely. It occupies either labia, but generally, by preference, the anterior, while not unfrequently, it festoons the entire cervix. This varicose condition of the cervix is sometimes transmitted to the uterus, in the form of general phlebitis, and when this occurs in the pregnant female, it invariably induces abortion. The discharge, from being glairy, at first, soon becomes brownish and purulent.

These forms of ulceration will require time for their removal, yet patience and proper treatment punctually given, will generally effect good results. The great practical point in treating granular ulceration of the os, is to regard it as merely an expression or manifestation of diseased action of the cervix, caused frequently, as before stated, by the passage of acrid discharges from parts above, and in turn, becoming itself, a secreting surface, inducing much annoyance and trouble, both by the profuse discharges, and the irritability and debility imparted to the constitution. The disease, is therefore, in a majority of cases, a symptom of another and graver condition existing within the cervix or uterus —a symptom requiring well-directed measures for its relief, while proper means, at the same time, are being used for the removal of the original cause of the disorder—such as cervical and corporeal endo-metritis—both by treatment addressed to those parts and to the system at large. Should exuberant granulations, or eversion of the cervical mucus membrane be discovered, they should be carefully removed; the first by scissors or the curette, the latter by paring the everted edges with the scissors, and approximating them, as accurately as possible, by sutures of silver wire.

*Follicular ulceration*, although not so common as the granular, yet is frequently observed. To fully understand the progress of this form of ulceration, it is essential that the fact be remembered that it results from an inflammatory action set up in the mucus follicles, which may be observed on the mucus membrane covering the vaginal surface of the cervix, as well as that lining the canal of the cervix and cavity of the uterus. These latter are lined by epithelium and basement membrane. "They contain a small quantity of mucus together with granular cells. Those upon, or near the margin of the os uteri" "sometimes contains short papillæ within their margin." "These glands secrete a clear, viscid, albuminous fluid, analagous to the prostatic fluid," constantly flowing from the neck, closing up the canal and covering the os, which, though a normal condition, has often been mistaken for a supposed catarrhal fluid.

Under inflammatory action, these glands are observed to become distended with fluid due to their retained secretions ; as this increases, the follicular parieties yield to the distending force, burst and pour out their contents, leaving minute points of depressed ulcerations at the site of follicular collapse, while the papillæ which they contain, very soon becomes hypertrophied, presenting small, red, elevated tubercles, hemorrhagic in appearance, instead of the depressed points of ulcerations. These various phases of follicular ulceration are often mistaken for apthæ, acne and herpes of the parts, and can only be distinguished from other forms of ulceration by a knowledge of the anatomical and physiological facts and pathological conditions upon which they depend.

This form of ulceration is caused from anything which may produce the granular variety, or that of cervical and corporeal endometritis ; and as these latter diseases often become exciting causes, in a majority of instances, they must be removed in order to effect a speedy or permanent cure—a result which is by no means probable or possible, without a knowledge of this fact. In treating this form of ulceration, in the early stage, before the follicles have been ruptured, they should be opened, and cauterized. After this stage, the treatment differs little from that demanded by the granular form, while, if, as happens in a majority of cases, it be complicated with cervical and corporeal endometritis, they should receive the treatment specially called for, by these diseased conditions.

*Syphilitic ulceration* is another form, which, if occurring in our practice, we have not been able to distinguish—save a few cases—from the other varieties. While, doubtless, the classical Hunterian chancre is, and may be, rarely observed as occurring on the cervix uteri, we think it more than probable that syphilitic ulceration must often present itself as occurring during the progress of secondary and tertiary syphilis—which, no doubt, are often confounded with that stage of ulceration presenting granulations, and known to systematic writers as granular ulcerations. Cases have come under our notice, differing, in no essential particulars, from the above, and which could not be classed as syphilitic—only so far as the existing fact of constitutional syphilis being present, and the ulceration yielding to general treatment for that malady, together with applications to the ulcer. This form of ulceration requires the same treatment applicable to chancre, with constitutional remedies in no way different from the usual and general means employed for secondary and tertiary forms of that disorder.

(*To be continued.*)

